# Editorial: Enhancing physical activity in women across the lifespan: evidence-based insights into quantification, intervention, outcomes, and limitations

**DOI:** 10.3389/fspor.2025.1699588

**Published:** 2025-10-08

**Authors:** Bojan Masanovic, Dušan Stupar, Szabolcs Halasi, Catalina Casaru, Gonul Babayigit Irez

**Affiliations:** ^1^ Faculty for Sport and Physical Education, University of Montenegro, Niksic, Montenegro; ^2^ Western Balkan Sport Innovation Lab, Podgorica, Montenegro; ^3^ Faculty of Sport and Psychology, TIMS, Educons University, Novi Sad, Serbia; ^4^ Hungarian Language Teacher Training Faculty, University of Novi Sad, Subotica, Serbia; ^5^ Department of Physical Education and Athletic Training, University of West Alabama, Livingston, AL, United States; ^6^ Faculty of Sport Sciences, Mugla Sitki Kocman University, Muğla, Türkiye

**Keywords:** women, public health, sports performance, physical activity interventions, physical fitness, monitoring systems, injury treatment

**Editorial on the Research Topic**
Enhancing physical activity in women across the lifespan: evidence-based insights into quantification, intervention, outcomes, and limitations

## 1 Introduction

Although sports and physical activity research has grown substantially over the past decades—with evidence from analyses of publications and citations demonstrating a marked increase in sports science over the last four decades, reflecting the growing recognition and importance of this field (1) — the research itself has predominantly focused on male participants, leaving a critical gap in our understanding of women's experiences. An analysis of publications indexed in Web of Science between 2014 and 2020 showed that 66% of study participants were men (8,253,236), whereas only 34% were women (4,254,445) (*p* < .0001), underscoring the persistent underrepresentation of women in sports and exercise science research (2). This imbalance constrains the development of knowledge regarding the unique physiological mechanisms, training loads, recovery patterns, and performance contexts specific to women (3). Addressing this gap is essential not only for advancing scientific understanding but also for informing practitioners, shaping effective interventions, and guiding policies that support women's health and performance. This collection directly addresses this gap by focusing on women's physical activity across different life stages and contexts, thereby expanding the evidence base that has historically been centered on men. Therefore, this Research Topic was created to further develop knowledge on women's physical activity across different life stages and to provide practical and theoretical insights that can contribute to science, policy, and practice.

## 2 Contribution to the field

The purpose of this Research Topic is to build a collection of new knowledge related to the effects of women’s physical activity across different life stages and to provide practical and theoretical insights that can contribute to science, policy, and practice. The 11 contributions (one Study Protocol and ten Original Research articles, including five cross-sectional studies, four randomized controlled trials, and one case study) offer readers an opportunity to substantially advance their knowledge in this field. In total, the sample included 3,082 participants (2,869 women and 213 men) from 12 countries (Serbia, Bulgaria, Greece, Romania, North Macedonia, China, South Korea, Spain, Czechia, Ghana, Poland, and Italy), ranging in age from 14 to 85 years old.

The studies included in this Research Topic span a wide spectrum of methodological approaches, themes, and age groups, which allowed for several possible classifications and interpretations. Ultimately, we decided to structure them into three thematic categories—(1) Gender and social factors, (2) Interventions and training programs, and (3) Specific populations and risks—and to interpret them accordingly.

The first group comprised four studies that addressed gender and social factors (one Study Protocol and three cross-sectional studies). The findings highlight the following: the development of a reliable protocol enabling the systematic monitoring of gender equality and women's representation in sports and sports leadership, with the potential to contribute to reducing the gender gap (Kascelan et al.); the significant influence of the built environment on the physical activity and mental well-being of young women in the Balkans, suggesting that inclusive urban planning could be a key strategy for improving public health (Masoumi et al.); that gender stereotypes reduce the likelihood of engaging in physical activity, whereas breaking these stereotypes and fostering a supportive environment may enhance women's participation in exercise (Guo & Huang); and that single parents who meet the WHO physical activity guidelines report a significantly better quality of life, with effects particularly pronounced among women and older adults (Owusu-Sarpong et al.).

The second group consisted of four studies evaluating various interventions and training programs, all of them randomized controlled trials. Based on their results, it can be concluded: that a six-week social support program in the form of the Mindful Mothering Nursing Intervention reduced parenting stress and improved parenting efficacy among mothers with adverse childhood experiences (Cho & Shin); that a 12-week high-intensity interval training program improved executive functions in young sedentary women, although changes in IGF-1 were not significant (Jimenez-Roldán et al.); that a 12-week taekwondo program increased muscle mass, enhanced physical function, and contributed to favorable metabolic changes in older sedentary women (Park et al.); and that a 12-week program of standard and Nordic walking in postmenopausal women improved exercise tolerance and induced favorable changes in body composition, with Nordic walking providing greater benefits for aerobic capacity and functional performance (Knappova et al.).

The third group included three studies that focused on specific populations and highlighted unique challenges (one case study and two cross-sectional studies). The results demonstrated that during a 5-week fight camp, the chronic and rapid weight loss experienced by an elite female Muay Thai athlete reduced her strength and basal metabolism, strained her kidney function, and worsened her blood parameters, pointing to health risks and the Relative Energy Deficiency in Sport (RED-s) syndrome. This finding also highlights the importance of individualized and evidence-based weight management strategies to mitigate negative health outcomes and enhance athletic performance in combat sports (Bulínová et al.); that handgrip strength (HGS) was only weakly to moderately associated with functional abilities and gait speed in older women, and therefore HGS is not sufficient as a stand-alone indicator of functional capacity, whereas chair stand tests (30s-CS and 5-CS) emerged as more reliable predictors of slow gait (Muollo et al.); and that higher accumulation of visceral fat (measured by MRI) is significantly associated with increased liver mass and unfavorable lipid and metabolic profiles, even when body weight, BMI, and total body fat were within normal ranges—indicating that visceral fat may represent a hidden risk even in healthy, young, non-obese adults (Zhang et al.).

## 3 Conclusion

This special issue, with its 11 articles, confirms the well-established evidence of the benefits of gender-sensitive research on physical activity and its potential to improve women's health and quality of life (4, Kascelan et al.). It further provides concrete proposals in the form of a wide spectrum of practical tools: psychological and social support programs for mothers; protocols for systematically monitoring gender equality in sports; various forms of structured physical activity programs designed for young, older, and postmenopausal women; validated tests for assessing functional capacity and the risk of slow gait in older adults; and methods for the early detection of hidden metabolic risks through visceral fat analysis. These tools are intended to serve the purposes of diagnosis, and the prevention of specific conditions, problems, and phenomena, in addition to their treatment, correction, or at least mitigation. Importantly, the proposed tools have been tested, and practitioners will be able to apply them either fully or partially, with a high probability of predicting transformation or the desired outcome.

Ultimately, we believe that the information and practical recommendations presented here will serve as a stimulus for new research ideas and innovative solutions, bringing us closer to more effective gender-sensitive strategies for improving women's health and performance in the future. These strategies are part of the ongoing endeavor to, paraphrasing the title of this research topic, enhance physical activity in women across the lifespan.

## References

[1] Wijaya A Al Ardha MA Nurhasan N Yang CB Lin RH Putro AB. Exploring the research trend and development of sports science technology in the last 4 decades: systematic review. Retos. (2024) 61:655–67. 10.47197/retos.v61.109306

[2] Cowley ES Olenick AA McNulty KL Ross EZ. “Invisible sportswomen”: the sex data gap in sport and exercise science research. Women Sport Phys Act J. (2021) 29(2):146–51. 10.1123/wspaj.2021-0028

[3] James JJ Klevenow EA Atkinson MA Vosters EE Bueckers EP Quinn ME Underrepresentation of women in exercise science and physiology research is associated with authorship gender. J Appl Physiol. (2023) 135(4):932–42. 10.1152/japplphysiol.00377.2023 37650136

[4] Strobl H Brew-Sam N Curbach J Metz B Tittlbach S Loss J. Action for men: study protocol of a community capacity building intervention to develop and implement gender-sensitive physical activity programs for men 50 plus. Front Public Health. (2020) 8:4. 10.3389/fpubh.2020.00004 32039133 PMC6992606

